# Prevalence of Misuse of Topical Corticosteroid among Dermatology Outpatients

**DOI:** 10.31729/jnma.5271

**Published:** 2020-11-30

**Authors:** Shristi Shrestha, Smita Joshi, Sajana Bhandari

**Affiliations:** 1 Department of Dermatology, Nepal Medical College and Teaching Hospital, Attarkhel, Kathmandu, Nepal

**Keywords:** *corticosteroids*, *dermatology*, *dermatophytoses*, *drug misuse*, *melasma*

## Abstract

**Introduction:**

Topical corticosteroids misuse has become one of the burning issues in many countries across the globe. They are known to cause a myriad of adverse effects which include local effects commonly and systemic effects rarely. In dermatology practice, one of the common problems we see these days are steroid-induced and steroid aggravated dermatoses. So, this study was done to find the prevalence of misuse of topical corticosteroid among dermatology outpatients.

**Methods:**

A descriptive cross-sectional study was done in the outpatient department of dermatology at a tertiary care hospital for 18 months. Ethical clearance was obtained from the Institutional Review Committee of NMCTH (Reference no. 029-076/077). Convenient sampling was done. Statistical Package for the Social Sciences (SPSS) version 16 was used to tabulate the data and analyze the results. Point estimate at 95% Confidence Interval was calculated along with frequency and proportion for binary data.

**Results:**

Out of 19464 patients, 614 (3.15%) (2.91%-3.39% at 95% Confidence Interval) gave a history of applying steroid containing creams. Among them, 220 (35.8%) belonged to the age group 21-30 years. Dermatophytoses were the skin disease where TCS was most commonly misused followed by melasma in 425 (69.2%) and 115 (18.7%) respectively. Beclomethasone was the steroid preparation that was misused in the maximum number of patients in 271 (44.1%). Some form of adverse effects was seen in 554 (88.6%) patients.

**Conclusions:**

Non-prescription sale of topical corticosteroids is the major cause of topical corticosteroids abuse in Nepal. Creating awareness among the prescribers as well as the patients is the current need.

## INTRODUCTION

TCS (Topical corticosteroids) is among the most commonly prescribed medication in dermatology in a wide variety of dermatoses including inflammatory, hyperproliferative and pruritic conditions.^1^ TCS are among the most commonly prescribed medication in dermatology. Depending upon their potency, TCS has been divided into different groups/classes.^2^ In almost all inflammatory dermatoses they provide rapid symptomatic relief. But their indiscriminate use can cause significant local adverse effects.^3^

TCS misuse has become one of the burning issues in many countries across the globe.^2,4–14^ Some of the contributing factors are non-prescription sales, lack of awareness, and non-availability of a qualified dermatologist.^8^ Similar to the global scenario, steroid-induced and steroid aggravated dermatoses are common in dermatology outpatient departments throughout Nepal.^15,16^ These conditions are more difficult to treat and cause much more distress to the patients than the primary conditions for which TCS were misused.

This study was therefore done to find the prevalence of patients misusing TCS among those visiting the dermatology outpatient department of a tertiary care hospital.

## METHODS

A descriptive cross-sectional study was conducted in the dermatology outpatient department of Nepal Medical College and Teaching Hospital, Kathmandu over 18 months from 1^st^ April 2018 to 30^th^ September 2019. Informed consent was taken from the patients. Ethical clearance was obtained from the Institutional Review Committee of NMCTH (Reference no. 029-076/077). Patients of any age and sex who consented to participate in the study were enrolled. Convenience sampling was done and the sample size was calculated using the formula,

$$\begin{array}{ll} \text{n} & \text{= }{\text{Z}}^{\text{2}}\times \text{p}\times \text{(1}-\text{p)}/{\text{e}}^{\text{2}}\\ & \text{= }{\left(1.96\right)}^{\text{2}}\times 0.5\times 0.5/{(\text{0.01)}}^{\text{2}}\\ & \text{= }9604 \end{array}$$

Where,

Z = 1.96 for 95% Confidence Interval
p = population proportion, 50%
e = margin of error, 1%

Since convenience sampling was used sample size was doubled to 19208. However, a total of 19464 patients were included in the study.

Statistical Package for the Social Sciences (SPSS) version 16 was used to tabulate the data and analyze the results.

## RESULTS

The total number of patients attending the dermatology OPD during this period was 19464. Of these, 614 (3.15%) of patients gave a history of applying steroid containing or other creams (ayurvedic/herbal/cosmetic) suspected to be containing steroid without indication. Out of these, 306 (49.8%) were male and 308 (50.2%) were female as shown in (Table 1).

**Table 1:** Primary dermatoses for which TCS containing products were applied.

Primary dermatoses	Male n (%)	Female n (%)	Total n (%)
Dermatophytosis	257 (84)	168 (54.5)	425 (69.2)
Melasma	11 (3.6)	104 (33.8)	115 (18.7)
Acne	3 (1)	10 (3.2)	13 (2.1)
Pityriasis versicolor	10 (3.3)	3 (1)	13 (2.1)
Scabies	10 (3.3)	3 (1)	13 (2.1)
Candidiasis	5 (1.6)	5 (1.6)	10 (1.6)
Eczema	2 (0.7)	8 (2.6)	10 (1.6)
Unknown	4 (1.3)	5 (1.6)	9 (1.5)
Others	4 (1.3)	2 (0.6)	6 (1.1)
Total	306 (100)	308 (100)	614 (100)

The youngest patient was 6 months old and the oldest was 76 years old with a mean age of 29.09 years (SD: 12.512). The maximum number of patients (35.8%) belonged to the age group 21-30 years.

The two most common indications where TCS misuse occurred were dermatophytoses 425 (69.2%) and melasma 115 (18.7%) (Table 1).

**Figure 1:**
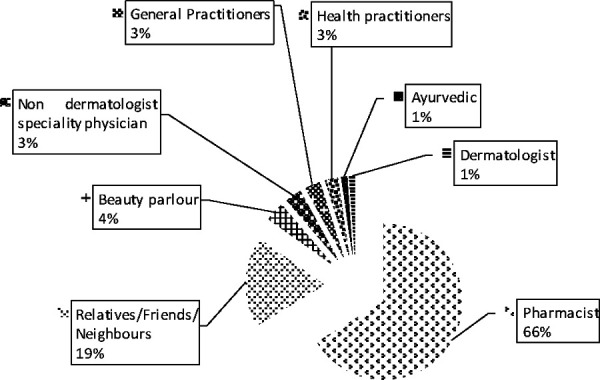
Persons who prescribed the TCS.

Regarding the prescriber of TCS, pharmacists were the ones who prescribed the most (66%) followed by relatives/friends/neighbors (19%) (Figure 1).

TCS is available in the market as a single component or in combination with other drugs. Steroid in combination with antifungal and antibiotic cream was the most common form used in 247 (58.1 %) patients with dermatophytoses whereas in melasma modified Kligman's formula containing steroid, hydroquinone, and tretinoin was misused by maximum patients 52 (45.2%) (Table 2).

**Table 2:** Commonly used drugs containing steroids in different indications.

Drug used	Dermatophytoses n (%)	Melasma n (%)	Others n (%)	Total n (%)
Steroid +Antifungal + Antibiotic (3 drugs)	247 (58.1)	9 (7.8)	28 (37.84)	284 (46.3)
Steroid only	59 (13.9)	38 (33)	23 (31.08)	120 (19.5)
Modified Kligman's formula	15 (3.5)	52 (45.2)	2 (2.7)	69 (11.2)
Unknown	50 (11.8)	5 (4.3)	3 (4.05)	58 (9.4)
Steroid + antifungal	22 (5.2)	0 (0)	9 (12.16)	31 (5)
Steroid + salicylic acid	17 (4)	3 (2.6)	3 (4.05)	23 (3.7)
Ayurvedic /Herbal	4 (0.9)	8 (7)	3 (4.05)	15 (2.4)
Steroid + Antibiotic	6 (1.4)	0 (0)	3 (4.05)	9 (1.5)
4 drugs combination	5 (1.2)	0 (0)	0 (0)	5 (0.8)

Beclomethasone dipropionate was the most commonly used TCS in 271 (44.1%) patients, followed by mometasone furoate in 107 (17.4%), clobetasol propionate in 86 (14%), and betamethasone valerate in 63 (10.3%) (Table 3).

**Table 3:** Commonly used steroids.

Drug used	Dermatophytoses n (%)	Melasma n (%)	Others n (%)	Total n (%)
Beclomethasone dipropionate	230 (54.1)	9 (7.8)	32 (43.24)	271 (44.1)
Mometasone furoate	31 (7.3)	67 (58.3)	9 (12.16)	107 (17.4)
Clobetasol Propionate	57 (13.4)	13 (11.3)	16 (21.63)	86 (14)
Betamethasone valerate	46 (10.8)	8 (7)	9 (12.16)	63 (10.3)
Unknown	50 (11.8)	5 (4.3)	3 (4.05)	58 (9.4)
Miscellaneous (Ayurvedic/Herbal)	4 (0.9)	8 (7)	3 (4.05)	15 (2.4)
Halobetasol propionate	3 (0.7)	0 (0)	2 (2.71)	5 (0.8)
Flucinolone acetonide	0 (0)	3 (2.6)	0 (0)	3 (0.5)
Fluticasone propionate	2 (0.5)	0 (0)	0 (0)	2 (0.3)
Hydrocortisone acetate	0 (0)	2 (1.7)	0 (0)	2 (0.3)
Triamcinolone acetonide	2 (0.5)	0 (0)	0 (0)	2 (0.3)

In dermatophytoses, beclomethasone was the steroid that was used by maximum patients, 230 (54.1%) whereas, in melasma, mometasone was the most commonly used steroid in 67 (58.3%) (Table 4). Out of the total 614 patients, 554 (88.6%) showed some form of adverse effects out of which 369 (70%) had only one, 140 (22.8%) had two, 44 (7.2%) had three, and one (0.2%) had four adverse effects. In dermatophytoses, tinea incognito/recurrence/extensive dermatophytoses were the commonest side effect whereas in melasma TSDF was the commonest side effect (Table 4).

**Table 4:** Prevalence of adverse effects of steroids.

Diseases Adverse effects	Dermato phytoses n (%)	Melasma n (%)	Others n (%)	Total n (%)
Tinea incognito/Recurrence/Extensive dermatophy toses	339(70.04)	0 (0)	37 (44.05)	376 (54.65)
TSDF	53 (10.95)	119 (99.17)	41 (48.81)	213 (30.96)
Striae	75 (15.5)	0 (0)	4 (4.76)	79 (11.48)
Secondary infection	17 (3.51)	1 (0.83)	2 (2.38)	20 (2.91)

## DISCUSSION

TCS has a valuable role in the management of a plethora of dermatoses and is amongst the most commonly prescribed medications from dermatologists. But the major issue globally at present is the issue of TCS misuse which has been shown in several studies done across the world.^2–14^ TCS provide dramatic relief from symptoms in many dermatology disorders which could be one of the reasons for rampant misuse. Besides in our country, it is easily available at a low cost over the counter and though there are regulatory laws in place preventing the sale of these drugs over the counter, the enforcement of these laws and monitoring of sales of TCS is almost nonexistent. Lack of awareness and non-availability of qualified dermatologists (especially in rural areas) are also contributing factors.

The prevalence of TCS misuse was 3.15% which was similar to an Indian study.8 In our study the most common age group affected was 21-30 years which was in accordance with other studies.^8,9,12^ In our study, males, and female were almost equal in number similar to a few past studies.^11–13^ Studies which include TCS use in facial skin show that the prevalence of misuse in the female is significantly high which may be because women are more conscious about the looks and melasma is commoner in females than males.^5–7,17–19,21,22^

TCS is misused in an ample number of dermatological diseases, but we have seen that dermatophytoses are the dermatoses where TCS is significantly misused which is similar to several other studies.^8–10,12^ The second most common indication for misusing TCS was melasma. The use of TCS on facial skin as a lightening agent has shown an alarming increase which has been depicted in many studies.^2,5–7,11,15–22^

Sixty-six percent of patients were using steroids on the advice of pharmacists. In 19% TCS use was suggested by relatives/friends/neighbors. In countries like Nepal and India, the easy availability of TCS over the counter at the chemist shops across the country without any valid prescription is compounding the problem of abuse which has been shown in multiple studies.^5,8,10,12,13,17,21^ TCS is available in the Nepalese market either alone or as a fixed drug combination (FDC) with antibiotic and antifungal. Steroid in combination with hydroquinone and tretinoin is also available as Kligman's or modified Kligman's formula (depending on the formulation of steroid used in the cream). Almost half (46.3%) of the total patients were using the FDC cream containing a steroid, antibiotic, and antifungal in our study. In patients with dermatophytoses, 58.1% were using this FDC which was similar to few studies.^8,12^ But in patients with melasma maximum (45.2%) were using modified Kligman's formula like in few Indian studies.^17,18,21^ When we considered the steroid formulation, beclomethasone was the most commonly used followed by mometasone and clobetasol. In many Indian studies, clobetasol propionate is the commonest steroid misused.^8,10,12,13^ In our study in dermatophytoses, beclomethasone was the most common steroid whereas, in melasma, mometasone topped the list of steroids. This is justified by the fact that in the Nepalese market most of the FDC creams available over the counter contain beclomethasone as a triple-drug combination and another two-drug combination (beclomethasone and clotrimazole) is also used very commonly as a drug for dermatophytoses over the counter. However, in melasma, the triple combination cream (modified Kligman's formula) which is available as a lightening agent contains mometasone as the steroid component in many brands. Besides, certain brands containing mometasone alone is also given by beauticians as a treatment for skin hyperpigmentation and to achieve general fairness and glow. In a Nepalese study done by Kumar et al, it was reported that beclomethasone dipropionate was the most commonly misused steroid over the face followed by mometasone furoate.^16^

Regarding the adverse effects of TCS misuse, different side effects were seen in patients with dermatophytoses and melasma. The most common adverse effect in patients with dermatophytosis was tinea incognito/ recurrence/extensive dermatophytoses. Tinea incognito also known as steroid modified tinea is a very common adverse effect of using TCS in dermatophytoses shown in many studies.^8–10,12^ TCS causes suppression of cutaneous inflammatory response which leads to ineffective elimination of the dermatophyte culminating in chronic and widespread infection.^23^

A new entity “TSDF” has been coined which is defined as “the semi-permanent or permanent damage to the skin of the face precipitated by the irrational, indiscriminate, unsupervised, or prolonged use of TCS resulting in a plethora of cutaneous signs and symptoms and psychological dependence on the drug”.^24^ In our study among people using TCS on the face for melasma, a significant percentage (99.17%) of patients had TDSF. TSDF includes various clinical features like erythema, papules, pustules (secondary bacterial infection), acneiform eruptions, hirsutism, telangiectasia, tinea incognito, hypo/hyperpigmentation, perioral dermatitis, rosacea like features, allergic contact dermatitis, photosensitivity, and atrophy/striae.^24^ Many studies have reported widespread abuse of TCS on the face and the common adverse effects mentioned are all part of TSDF.^5,7–10,16–22^

Prolonged use of TCS (mostly high and ultra-high potency) can cause systemic adverse effects though lesser than systemic corticosteroids mostly in infants and early.^25^ Bhusal M et al reported a 32-year-old female who developed iatrogenic Cushing's syndrome following the application of clobetasol propionate for disseminated tinea infection.^26^ However in our study we did not find any systemic adverse effects.

Our study is consistent with the observation that factors leading to misuse of TCS is at various stages from manufacturer to prescriber to salesperson to layperson.^4^

Our study is limited as the sample is restricted to one outpatient department in one location. A multicentre study across the country is recommended to find out the true magnitude of this multifaceted problem of TCS misuse. The adverse effects of TCS could be elaborated further by specifying the different types of lesions seen in tinea incognito and in TSDF which could help us determine the commoner manifestations in our skin type.

## CONCLUSIONS

The misuse of TCS has been increasing rampantly in recent years. TCS has been used as “steroid cocktails” mostly in dermatophytoses which have to lead to the emergence of the issue of resistant dermatophytoses. Besides the use of TCS as fairness cream and as a treatment for hyperpigmentation is also pretty common which has lead to the increasing occurrence of TSDF. Factors contributing to this epidemic are pharmaceutical industry manufacturing and promoting inappropriate FDC containing TCS, unregulated over the counter supply and unrestricted sale of TCS, professional ignorance (qualified and unqualified persons prescribing TCS), and public ignorance (layperson using it themselves or advising friends/family to use it). So it is high time we raise public awareness and educate the prescribers (chemists and medical practitioners). Another major step would be to make the government aware of this issue and regulate the manufacture and over the counter sale of these steroid combinations. Topical steroids and their combinations need to be sold as “prescription-only” drugs.
